# Lifestyle factors and quality of life among primary health care physicians in Madinah, Saudi Arabia

**DOI:** 10.1016/j.sjbs.2021.04.087

**Published:** 2021-05-04

**Authors:** Atallah Mohammad Aljohani, Abdulmohsen Hamdan Al-Zalabani

**Affiliations:** a Ministry of Health, General Health and Preventive Medicine Administration, Preventive Medicine Clinics Complex, Madinah, Saudi Arabia; b Taibah University, Department of Family and Community Medicine, College of Medicine, Madinah, Saudi Arabia

**Keywords:** Lifestyle, Quality of life, Health behavior, Physicians, Primary health care, Saudi Arabia

## Abstract

**Background:**

Physicians are considered to be a high-risk population for a poor quality of life (QoL), but few studies of lifestyle factors include the QoL among them.

**Objectives:**

This study aimed to investigate the relationship between lifestyle factors and a positive QoL among primary health care (PHC) physicians.

**Methods:**

A cross-sectional study was conducted at 20 primary healthcare centers in Madinah, Saudi Arabia. A self-administered questionnaire was used, including sociodemographic characteristics, lifestyle data, and the short version of the World Health Organization Quality of Life questionnaire. Appropriate statistical analyses were used, including multivariate logistic regression models.

**Results:**

The response rate was 85.7% (72/84) physicians. The mean score of the total QoL and its four studied domains (physical, psychological, social, and environmental) was relatively high, with no statistically significant difference between the consultants and general practitioners. The positive total QoL in this study was significantly lower among physicians with obesity (OR = 0.55, 95%CI = 0.25–0.97), those using butter and animal fat for cooking (OR = 0.10, 95%CI = 0.02–0.81), and those eating meals out > 3 times per week (OR = 0.30, 95%CI = 0.10–0.90). Although non-significant, vegetable consumption and a high level of physical activity were associated with a positive QoL, with adjusted ORs of 2.5 (95%CI = 0.82–7.58) and 1.5 (95%CI = 0.33–6.65), respectively.

**Conclusion:**

The findings indicate a relatively good QoL among the participating physicians; however, a lower QoL was associated with unhealthy lifestyle factors. QoL was significantly associated with obesity, cooking practices, and eating meals from restaurants.

## 1 Introduction

There has been great concern about the role of lifestyle factors in preventing chronic diseases, and their role in preventive medicine. Along with tobacco use, nutrition, the body mass index (BMI), and physical activity are the main factors affecting the quality of life (QoL) that can be modified through lifestyle/behavioral changes (Renehan and Howell, 2005, WHO, 2018). Regionally, recent studies have stressed the impact of lifestyle factors on the QoL among patients with different chronic diseases (Aboshaiqah et al., 2016, Qusaier et al., 2016).

Concerning the effects of these factors on the QoL of health care workers, most published studies are from the Western world, stressing mainly the impacts of work and environmentally-related factors on job burnout (Cimiotti et al., 2012, Galletta et al., 2016). Few studies have discussed the health-related quality of life (HRQoL) among certain medical specialties, such as psychiatrists (Liu et al., 2015) and anesthesiologists (Arenson-Pandikow et al., 2012).

Physicians, in general, are considered to be a high risk population for a poor QoL. They spend practically all of their time in a state of alert while practicing medicine. Patient care and unexpected work situations generate a gradual and increasing overload among them. The literature confirms considerable contributions supporting the prevalence of psychophysiological alterations among physicians (Haber et al., 2013, Hunsaker et al., 2015). However, there is still a lack of studies regarding the other aspects of the QoL and its related lifestyle risk factors among primary health care (PHC) physicians, particularly in Saudi Arabia. PHC services provide a large part of the basic health care to the Saudi community, with >80% of the total visits to the Ministry of Health facilities occurring in PHC centers (PHCCs) (Ministry of Health, 2016). Therefore, studies that assess the QoL among PHC physicians and its related risk factors are very important in light of improving PHC services and physician performance.

The present study aimed to investigate the relationship between the known lifestyle factors and a positive QoL among PHC physicians in Madinah, Saudi Arabia.

## 2 Subjects and methods

### 2.1 Study setting, and population

This cross-sectional study recruited health care workers from PHCCs in Madinah, Saudi Arabia, from December 2015 to August 2016. It was designed to assess the QoL among the study population and to investigate the associations between a positive QoL and known lifestyle risk factors.

Madinah is located in the western region and is the fourth largest city in Saudi Arabia. It is the capital of the Al Madinah province, with a total provincial population of 1.7 million people. Madinah is divided into four health sectors belonging to the Ministry of Health, each consisting of 9–11 PHCCs. In total, 173 physicians and 554 nurses work at these centers (Ministry of Health, 2016).

### 2.2 Sampling procedures

A cluster random sampling procedure was used. Twenty PHCCs (clusters) were selected using the probability proportion to size (PPS) random sampling technique from the list of all PHCCs in Madinah. In each selected PHCC, all of the working physicians were invited to participate in the study. All physicians regardless of sex, training level or nationality were eligible to participate.

### 2.3 Data collection instruments

The data were collected by using a self-administered paper-based questionnaire that was distributed to physicians in each center. The questionnaire was divided into three sections. The first section included the sociodemographic data (age, sex, marital status, occupational title, and occupational years). The second section included data about health-related lifestyle factors (smoking, BMI, cooking oil, meals out per week, total fruit and vegetable intake per day, physical activity level, and hours of TV, laptop, or internet use per day). The questions on lifestyle factors were adopted from the World Health Organization (WHO) STEPwise approach to surveillance (STEPS) survey that was used previously in a study carried out by the Saudi Ministry of Health (Al-Zalabani et al., 2015, WHO, 2005, WHO, xxxx).

The physical activity information included questions about activities at work, travel to and from places, recreational activities, and sedentary behavior. The total physical activity was calculated according to the STEPS manual (World Health Organization, n.d.), and then categorized into three levels (low, moderate, and high), in which a high level corresponded to vigorous activity on at least 3 days per week or combination of moderate or vigorous intensity on at least 7 days; moderate level corresponded to moderate activity on at least 5 days per week or combination of moderate or vigorous intensity on at least 5 days; and low level corresponded to physical inactivity where criteria for high and moderate levels were not achieved. The BMI, as a measure of overweight and obesity, was calculated as the division of the self-reported weight in kilograms by the height in meters squared (kg/m^2^). Obesity was defined for both sexes as a BMI of ≥ 30 kg/m^2^, with overweight as a BMI of 25 to < 30 kg/m^2^, according to the WHO (The World Health Organization, 2000).

The last section of the questionnaire included QoL data collected using the Arabic short version of the WHO Quality of Life (WHOQOL-BREF) scale, which included 26 questions (“Development of the World Health Organization WHOQOL-BREF quality of life assessment. The WHOQOL Group.,” 1998). The instrument showed a good internal consistency in the current study with Cronbach's alpha of 0.90.

The QoL was the dependent variable that was assessed by the WHOQOL-BREF using four domains: physical (7 items), psychological (8 items), social (3 items), and environmental (8 items). The scores of the domain items denote the subject's perception of the QoL in each particular domain.

The domain scores were scaled in a positive direction, with higher scores denoting higher QoLs. The study considered a high QoL to be a good quality (positive quality), with a cut-off point for the QoL of 65% or above. This cut-off point selection was based on a previous study (Bani-Issa, 2011). The average score of each domain item was used to calculate the domain score. The average scores were then multiplied by four to make the domain scores comparable to those of the WHOQOL-100. In this way, they were changed to a scale of 0–100. The total QoL and domain scores were then computed and reported.

### 2.4 Statistical analysis

The data collected from the participating physicians was analyzed using the statistical analysis system (SAS) software package (SAS Institute Inc., 1999). The physicians' characteristics were tabulated so that the categorical variables were presented by the frequency number and percent, and the continuous variables were presented by the mean ± SD. The mean scores of the total QoL and its aspects were compared among the physicians by their job titles using an independent *t*-test. A p value ≤ 0.05 was considered to be an indicator of a statistically significant difference. Multivariate logistic regression analyses were performed to examine the association of the QoL and its domains with the studied lifestyle risk factors, while controlling for possible confounding factors. The potential confounders included in all of the analyses were age (<32 and ≥ 32 years old), sex (male vs. female), job title (consultants and specialists vs. general practitioners), marital status (singe, married, widow, and divorced), and occupational years.

### 2.5 Ethical considerations

Approval was obtained from the local Ethics Committee at the Department of Public Health in Madinah, Saudi Arabia. Each participant signed a written informed consent. Best practices were followed to ensure the confidentiality and privacy of the collected data.

## 3 Results

A total of 72 PHC physicians (43 males and 29 females) were analyzed to examine the association between their lifestyle factors and QoLs. This represented a response rate of 85.7% (72 out of 84 physicians invited to participate). Table 1 presents the sociodemographic characteristics of the participating physicians. Their mean age was 34.4 ± 8.6 years old (25–61), and most of them were married (72.2%). The consultants and specialists made up 37.5% of this cohort, while the general practitioners made up 62.5%, with mean occupational years of 5.3 ± 5.2 (2–30). About 24% of the physicians (n = 17) complained of chronic diseases related to their lifestyle risk factors.

**Table 1:** Characteristics of the studied physicians, Madinah, Saudi Arabia.

Characteristics*	N = 72
**Age in years,** mean ± SD (range)	34.4 ± 8.6 (25–61)
**Age in years** <32 ≥32	33 (45.8) 39 (54.2)
**Sex** Male Female	43 (59.7) 29 (40.3)
**Marital status** Single Married Widow and divorced	14 (14.8) 52 (72.2) 6 (8.3)
**Job title** Consultants and specialists General practitioners	27 (37.5) 45 (62.5)
**Occupational years,** mean ± SD (range)	5.3 ± 5.2 (2–30)
**Lifestyle medical related problems** No Yes	55 (76.4) 17 (23.6)

* Data are presented by the mean ± SD or by n (%).

Table 2 presents the mean QoL scores of the physicians by their job titles (consultants and specialists vs. general practitioners). The total mean QoL score was slightly higher in the general practitioners (287.6 ± 44.7) when compared to that of the consultants and specialists (286.7 ± 46.4), although this was not statistically significant. When addressing the different aspects of the QoL, the psychological QoL score was also slightly higher in the general practitioners. The mean scores of the physical and social aspects of the QoL, however, were slightly higher among the consultants and specialists when compared to the general practitioners, but no statistically significant difference was detected. The mean score of the environmental QoL was nearly the same in both groups.

**Table 2:** Quality of life scores of the primary health care physicians by their job title, Madinah, Saudi Arabia*

	Consultants and specialists (n = 27)	General practitioners (n = 45)	P value
	Mean ± SD	Mean ± SD	
Total quality of life scores	286.7 ± 46.4	287.6 ± 44.7	0.93
Physical quality of life scores	79.1 ± 13.9	77.9 ± 10.1	0.73
Psychological quality of life scores	70.5 ± 9.9	71.2 ± 10.8	0.83
Social quality of life scores	74.7 ± 13.9	73.8 ± 17.3	0.83
Environmental quality of life scores	67.9 ± 10.9	67.9 ± 10.5	0.99

* n: number; SD: standard deviation.

The association between the lifestyle factors and positive total QoL in the PHC physicians is shown in Table 3. A significant reduction in the positive total QoL was found in the obese physicians (OR = 0.55, 95%CI = 0.25–0.97), and those who reported eating meals out > 3 times per week (OR = 0.30, 95%CI = 0.10–0.90). A markedly significant reduction of 90% was also detected among those physicians using butter and animal fat in cooking, with an adjusted OR of 0.10 (95%CI = 0.02–0.81). Sitting in front of the TV, laptop, or internet for>3 h per day was associated with an insignificant reduction in the positive QoL of 60%, with an adjusted OR of 0.40 (95%CI = 0.10–1.45). However, eating > 3 vegetable servings per day and practicing a high level of physical activity were associated with an insignificant increase in the positive QoL, with adjusted ORs of 2.5 (95%CI = 0.82–7.58) and 1.5 (95%CI = 0.33–6.65), respectively. The other lifestyle factors studied appeared to have no observable effect on the positive QoL among the studied physicians.

**Table 3:** Association of lifestyle factors with positive quality of life among primary health care physicians, Madinah, Saudi Arabia.

Lifestyle factors	Positive QoL* (n = 51)	Negative QoL (n = 21)	OR**	95% CI
**Smoking** Never and Ex-smokers Current smokers	40 11	17 4	1.00 1.15	Ref. 0.78–3.10
**Body mass index (kg/m^2^)** <25 25–<30 ≥30	22 20 9	6 7 8	1.00 0.45 0.55	Ref. 0.15–1.93 0.25–0.97***
**Cooking oil** Vegetable oil Butter and animal fat	50 1	17 4	1.00 0.10	Ref. 0.02–0.81***
**Meal outside home/week** **≤**3 >3	24 27	4 17	1.00 0.30	Ref. 0.10–0.90***
**Total items of fruit intake/day** **≤**3 >3	29 22	11 10	1.00 1.20	Ref. 0.40–3.27
**Total items of vegetables intake/day** ≤ 3 >3	21 30	11 10	1.00 2.50	Ref. 0.82–7.58
**Physical activity levels** Low Moderate High	16 16 19	66 11 4	1.00 0.25 1.50	Ref. 0.10–1.05 0.33–6.65
**Sitting hours in front of TV, laptop, internet, etc./day** **≤**3 h >3 h	43 8	15 6	1.00 0.40	Ref. 0.10–1.45

* Positive quality of life includes those subjects whom domain score exceeds 65%. Negative QoL is the reference group

** OR are adjusted for age, sex, job title, occupational years, marital status and chronic diseases.

*** Significant.

Table 4 displays the association of the lifestyle factors with the positive physical and psychological aspects of the QoL among the PHC physicians. Although not significant, the positive physical QoL was found to decrease with smoking, butter and animal fat cooking, overweight and obesity, moderate and high levels of physical activity, and sitting > 3 h in front of the TV, internet, or laptop. A significant reduction was observed among those physicians who reported eating out>3 times per week, with an adjusted OR of 0.25 (95%CI = 0.10–0.97). The positive physical QoL, however, was found to increase 3 (95%CI = 1.01–9.98) and 1.5 (95%CI = 0.50–4.70) times among those physicians who reported eating >3 servings of fruit and vegetables, respectively. The positive psychological QoL was found to insignificantly increase in association with smoking (OR = 1.90, 95%CI = 0.40–9.20) and moderate (OR = 1.50, 95%0.40–5.60) and high (OR = 1.40, 95%CI = 0.40–5.20) levels of physical activity. Although not significant, a positive psychological QoL was reduced by overweight and sitting > 3 h in front of the TV, internet, or laptop. The adjusted odds ratios were 0.6 (95%CI = 0.20–2.03) and 0.5 (95%CI = 0.20–1.80), respectively.

**Table 4:** Association of lifestyle factors with positive physical and psychological aspects of quality of life among primary health care physicians, Madinah, Saudi Arabia.

Lifestyle factors	Physical QoL*		Psychological QoL*	
	+ve/−ve 53/19	OR** (95%CI)	+ve/−ve 50/22	OR** (95%CI)
**Smoking** Never and Ex-smokers Current smokers	47/16 6/3	1.0 (Ref.) 0.50 (0.10–2.38)	42/20 8/2	1.0 (Ref.) 1.90 (0.40–9.20)
**Body mass index (kg/m^2^)** <25 25–<30 ≥30	23/5 18/9 12/5	1.0 (Ref.) 0.30 (0.10–1.10) 0.60 (0.30–1.34)	20/8 17/10 13/4	1.0 (Ref.) 0.60 (0.20–2.03) 1.15 (0.60–2.40)
**Cooking oil** Vegetable oil Butter and animal fat	51/16 2/3	1.00 (Ref.) 0.15 (0.10–1.21)	48/19 2/3	1.0 (Ref.) 0.30 (0.05–2.17)
**Meal outside home/week** **≤** 3 > 3	25/3 28/16	1.0 (Ref.) 0.25 (0.10–0.97)	20/8 30/12	1.0 (Ref.) 0.99 (0.35–2.90)
**Total items of fruit intake/day** ≤ 3 >3	26/13 26/6	1.0 (Ref.) 3.00 (1.01–9.98)	28/12 22/10	1.0 (Ref.) 1.04 (0.36–2.97)
**Total items of vegetables intake/day** ≤ 3 > 3	30/12 23/7	1.0 (Ref.) 1.50 (0.50–4.70)	32/10 18/12	1.0 (Ref.) 0.50 (0.20–1.37)
**Physical activity levels** Low Moderate High	18/4 19/8 16/7	1.0 (Ref.) 0.55 (0.14–2.10) 0.50 (0.13–2.06)	14/8 19/8 17/6	1.0 (Ref.) 1.50 (0.40–5.60) 1.40 (0.40–5.20)
**Sitting hours in front of TV, laptop, internet, etc./day** ≤ 3 h > 3 h	44/14 9/5	1.00 0.60 (0.20–2.36)	42/16 8/6	1.0 (Ref.) 0.50 (0.20–1.80)

* Positive environmental aspects of quality of life include those subjects whom domain score exceeds 65%.

** OR are adjusted for age, sex, job title, occupational years, marital status, and chronic diseases.

The associations between the lifestyle factors and positive social and environmental aspects of the QoL in the studied physicians can be seen in Table 5. A positive social QoL was found to decrease among smokers, overweight and obese physicians, those eating out > 3 times per week, and those sitting > 3 h in front of the TV, internet, or laptop, with a markedly significant reduction of about 90% detected among those physicians reporting the use of butter and animal fat in cooking, with an adjusted OR of 0.10 (95%CI = 0.03–0.47). A total vegetable intake of >3 items per day, however, was found to increase the positive social QoL among the physicians (OR = 1.5, 95%CI = 0.87–3.89). Although insignificant, the positive environmental QoL was increased by 1.7 (95%CI = 0.65–4.45) among the physicians reporting an intake of >3 fruit items per day, and 1.9 (95%CI = 0.60–6.5) among those practicing a high level of physical activity. The adjusted ORs for the other lifestyle factors studied indicated an insignificant reduction in the positive environmental QoL among physicians with overweight or obesity, those reporting eating out > 3 meals per week, and among those sitting > 3 h in front of the TV, internet, or laptop.

**Table 5:** Association of lifestyle factors with positive social and environmental aspects of quality of life among primary health care physicians, Madinah, Saudi Arabia.

Healthy lifestyle factors	Social QoL*		Environmental QoL*	
	+ve/−ve 61/11	OR** (95%CI)	+ve/−ve 41/31	OR** (95%CI)
**Smoking** Never and Ex-smokers Current smokers	52/9 9/2	1.0 (Ref.) 0.80 (0.47–2.10)	35/28 6/3	1.0 (Ref.) 1.40 (0.30–6.50)
**Body mass index (kg/m^2^)** <25 25–<30 ≥30	25/3 23/4 13/4	1.0 (Ref.) 0.45 (0.10–2.60) 0.48 (0.20–1.21)	19/9 13/14 9/8	1.0 (Ref.) 0.40 (0.11–1.15) 0.70 (0.40–1.30)
**Cooking oil** Vegetable oil Butter and animal fat	59/8 2/3	1.0 (Ref.) 0.10 (0.03–0.45)	39/28 2/3	1.0 (Ref.) 0.40 (0.10–3.25)
**Meal outside home/week** **≤** 3 > 3	26/2 35/9	1.0 (Ref.) 0.30 (0.10–1.60)	18/10 23/21	1.0 (Ref.) 0.70 (0.25–1.75)
**Total items of fruit intake/day** **≤** 3 >3	37/7 24/4	1.0 (Ref.) 1.15 (0.49–3.50)	21/19 20/12	1.0 (Ref.) 1.70 (0.65–4.45)
**Total items of vegetables intake/day** ≤ 3 > 3	39/8 22/3	1.0 (Ref.) 1.50 (0.87–3.89)	25/17 16/14	1.0 (Ref.) 0.90 (0.30–2.10)
**Physical activity levels** Low Moderate High	20/2 20/7 21/2	1.0 (Ref.) 0.20 (0.05–1.10) 1.20 (0.15–10.1)	12/10 13/14 16/7	1.0 (Ref.) 0.90 (0.20–2.50) 1.90 (0.60–6.50)
**Sitting hours in front of TV, laptop, internet, etc./day** **≤** 3 h > 3 h	51/7 10/4	1.0 (Ref.) 0.40 (0.10–1.60)	34/24 7/7	1.0 (Ref.) 0.70 (0.25–2.50)

* Positive environmental aspects of quality of life include those subjects whom domain score exceeds 65%.

** OR are adjusted for age, sex, job title, occupational years, marital status, and chronic diseases.

## 4 Discussion

The QoL includes a wide multidimensional concept that is usually concerned with a subjective assessment of all aspects of life (The World Health Organization Quality of Life Assessment (WHOQOL)., 1998). Several factors can influence an individual’s perception of the QoL, including lifestyle factors. Since physicians are considered to be a high-risk population for a poor QoL, the present study aimed to examine the association between lifestyle factors and the QoL among PHC physicians in Madinah, Saudi Arabia.

The study findings revealed no significant differences in the scores of the mean total QoL and its aspects between the consultants and general practitioners. The same findings were also reported in a cross-sectional study conducted in Brazil, in which the overall WHOQOL-BREF score and its domains (physical, psychological, social, and environmental) did not show any statistically significant differences between the studied anesthesiologists, non-anesthesiologists, and physicians working in different health care facilities (Arenson-Pandikow et al., 2012).

Although not significant, the mean physical QoL in the present study was higher among the consultants and specialists when compared with the general practitioners. It was 79.1 ± 13.9 among the consultants and specialists and 77.9 ± 10.1 among the general practitioners. Because of their experience in the diagnosis and treatment of diseases, consultants and specialists may take better care of themselves physically, and be more likely to respond well to any changes in their physical health (Liu et al., 2015). This could explain their relatively better physical QoL when compared with that of the general practitioners.

The proportion of PHC physicians with a positive (good) QoL was relatively high in the present study. It was 70.8% for the total QoL, 73.6% for the physical QoL, 69.4% for the psychological QoL, 84.7% for the social QoL, and 56.9% for the environmental QoL. Liu et al. (Liu et al., 2015) reported a good physical QoL but impaired mental QoL among Chinese psychiatrists. The high level of psychological stress, job burnout, and the job environment, may contribute to the poor mental health among psychiatrists (Firth-Cozens, 2007, Rathod, 2000). The presentation of the psychiatrists in the present study sample, however, was negligible. Moreover, the variation between countries regarding the health system, number of working hours, rumination, annual leaves, and other related factors may explain the differences in QoL estimates among physicians in different countries. With respect to the physical QoL, some reports have suggested inadequate physical health care among physicians of different specialties (Kay et al., 2004, Linn, 1985) when compared with the general population. However, other studies have reported a low mortality rate among physicians (Schlicht et al., 1990), since they are more likely to practice infection control and prevention (Chambers, 1992).

The positive total QoL in this study was reduced among physicians with obesity, those reporting eating out > 3 times per week, and those who sat > 3 h per day in front of a TV, laptop, or internet. A marked reduction of 90% was also detected among those physicians using butter and animal fat in cooking. Vegetable consumption and a high level of physical activity were associated with a better QoL, with adjusted ORs of 2.5 and 1.5, respectively. Obesity is known to influence health, in general, and it is considered to be an important risk factor for several diseases (The World Health Organization, 2000); as such, it has been reported to affect the HRQoL. Furthermore, a modest weight reduction as a result of lifestyle modification has appeared to ameliorate problems with the HRQoL (Fontaine and Barofsky, 2001). Butter and animal fat cooking is a risk factor for developing atherosclerosis, hypertension, and cardiovascular disease. The results of a recent study in China carried out on 775 scientific workers reported higher physical HRQoL scores among those reporting a daily intake of vegetable oils when compared with those reporting a daily intake of animal fat (Gong et al., 2015).

Physical activity and good dietary habits are indicators of a good HRQoL (WHO, 2018). At the Mayo Clinic in Rochester, Minnesota, an incentivized exercise program survey was carried out to study the effects of physical exercise on the QoL and burnout among physician trainees (Weight et al., 2013). When compared to the nonparticipants, the study results revealed a significantly higher QoL in the program participants (median 68 vs 75, p < 0.001). Although not significant, the burnout was also lower in the program participants when compared with the nonparticipants (Weight et al., 2013). A recent study in the USA on 3594 individuals aged 60 years old and older reported significant associations between physical exercise and self-reported health and physical and mental health days. In addition, the study found significant associations between the fruit and vegetable consumption and physical health days (Kwon et al., 2015).

The abovementioned lifestyle factors were found to affect each of the domains of the QoL (psychological, physical, social, and environmental) in a way similar to that of the total QoL, but with some variations in the characteristics for each domain.

The present study strengths include the fact that the study questionnaire used was comprehensive, and the assessment of the HRQoL was based on a simple and valid WHOQOL-BREF (“Development of the World Health Organization WHOQOL-BREF quality of life assessment. The WHOQOL Group.,” 1998). The lifestyle factors included in the study questionnaire were adopted from the WHO STEP survey, which was used previously in a study done by the Saudi Ministry of Health (WHO, 2005). To the best of our knowledge, the present study is the first to investigate the association between the QoL and its domains and lifestyle factors among PHC physicians in Saudi Arabia.

One limitation was that this study was cross-sectional in nature; thus, the causal relationships could not be demonstrated between the observed associations. Although the study achieved a high response rate, the generalization of the study results was difficult to apply to other physicians in different settings due partly to the sociocultural variations and partly to the small sample size. Self-selection and information bias may represent another limitation factor in this study. However, the high response rate and anonymous and self-administered questionnaire have greatly minimized such expected bias.

## 5 Conclusion

In conclusion, the present findings indicate relatively good QoLs in the participating PHC physicians. However, the QoL can be substantially impaired with unhealthy lifestyle factors. In the present study, QoL was significantly associated with obesity, cooking practices, and eating meals from restaurants. From a clinical perspective, the QoL among PHC physicians should be considered in PHC monitoring in Saudi Arabia to estimate its burden on the quality of health care services provided to the Saudi community. Further research to explore the determinants of the QoL in PHC physicians in a large national sample from different Saudi regions is warranted.

## Funding

This research did not receive any specific grant from funding agencies in the public, commercial, or not-for-profit sectors.

## Declaration of Competing Interest

The authors declare that they have no known competing financial interests or personal relationships that could have appeared to influence the work reported in this paper.

## References

[b0005] Aboshaiqah A., Al-Saedi T.S.B., Abu-Al-Ruyhaylah M.M.M., Aloufi A.A., Alharbi M.O., Alharbi S.S.R., Al-Saedi A.S., Al-Erwi A.F. (2016). Quality of life and satisfaction with care among palliative cancer patients in Saudi Arabia. Palliat. Support. Care.

[b0010] Al-Zalabani A.H., Al-Hamdan N.A., Saeed A.A. (2015). The prevalence of physical activity and its socioeconomic correlates in Kingdom of Saudi Arabia: A cross-sectional population-based national survey. J. Taibah Univ. Med. Sci..

[b0015] Arenson-Pandikow H.M., Oliviera L.T., Bortolozzo C.R., Petry S., Schuch T.F. (2012). Perception of quality of life among anesthesiologists and non-anesthesiologists. Rev. Bras. Anestesiol..

[b0020] Bani-Issa W. (2011). Evaluation of the Health-Related Quality of Life of Emirati People with Diabetes: Integration of Sociodemographic and Disease-Related Variables. East. Mediterr. Heal. J..

[b0025] Chambers R. (1992). Health and lifestyle of general practitioners and teachers. Occup Med (Lond)..

[b0030] Cimiotti J.P., Aiken L.H., Sloane D.M., Wu E.S. (2012). Nurse staffing, burnout, and health care-associated infection. Am. J. Infect. Control.

[b0035] Development of the World Health Organization WHOQOL-BREF quality of life assessment. The WHOQOL Group, 1998. Psychol. Med., vol. 28, pp. 551–558. 10.1017/s0033291798006667. 9626712

[b0040] Firth-Cozens J. (2007). Improving the health of psychiatrists. Adv Psychiatr Treat.

[b0045] Fontaine K.R., Barofsky I. (2001). Obesity and health-related quality of life. Obes. Rev..

[b0050] Galletta M., Portoghese I., Ciuffi M., Sancassiani F., Aloja E.D., Campagna M. (2016). Working and environmental factors on job burnout: a cross-sectional study among nurses. Clin. Pract. Epidemiol. Ment. Heal..

[b0055] Gong Q., Tu L., Zhou L., Chen H. (2015). Associations between Dietary Factors and Self-Reported Physical Health in Chinese Scientific Workers. Int. J. Environ. Res. Public Health.

[b0060] Haber Y., Palgi Y., Hamama-Raz Y., Shrira A.B.-E.M. (2013). Predictors of professional quality of life among physicians in a conflict setting: the role of risk and protective factors. Isr J. Psychiatry Relat. Sci..

[b0065] Hunsaker S., Chen H.C., Maughan D., Heaston S. (2015). Factors that influence the development of compassion fatigue, burnout, and compassion satisfaction in emergency department nurses. J. Nurs. Scholarsh..

[b0070] Kay, M.P., Mitchell, G.K., Del Mar, C.B., 2004. Doctors do not adequately look after their own physical health. Med. J. Aust. https://doi.org/kay10461_fm [pii]. 10.5694/j.1326-5377.2004.tb06329.x 15462653

[b0075] Kwon S.C., Wyatt L.C., Kranick J.A., Islam N.S., Devia C., Horowitz C., Trinh-Shevrin C. (2015). Physical activity, fruit and vegetable intake, and health-related quality of life among older Chinese, Hispanics, and blacks in New York City. Am. J. Public Health.

[b0080] Linn L.S. (1985). Health status, job satisfaction, job stress, and life satisfaction among academic and clinical faculty. JAMA J. Am. Med. Assoc..

[b0085] Liu C., Wang L., Zhao Q. (2015). Factors related to health-related quality of life among Chinese psychiatrists: occupational stress and psychological capital. BMC Health Serv. Res..

[b0090] Ministry of Health (2016). Statistical Yearbook.

[b0095] Qusaier R.A., Al-Tqiqi Y.E., Al-Ahmadi H.S., Felemban A.M.A.-Q.A. (2016). Quality of life among hypertensive patients attending primary healthcare centers in Jeddah, Saudi Arabia. Int. J. Adv. Res..

[b0100] Rathod S. (2000). A survey of stress in psychiatrists working in the Wessex Region. Psychiatr. Bull..

[b0105] Renehan A.G., Howell A. (2005). Preventing cancer, cardiovascular disease, and diabetes. Lancet.

[b0110] SAS Institute Inc., 1999. Proprietary Software Release 8.2. Cary, NC, SAS Institute Inc., 1999.

[b0115] Schlicht S.M., Gordon I.R., Richard J., Ball B., Christie D.G.S. (1990). Suicide and related deaths in Victorian doctors. Med. J. Aust..

[b0120] The World Health Organization, 2000. Obesity: Preventing and Managing the Global Epidemic- Report of a WHO consultation, WHO Tech Rep Ser. 11234459

[b0125] The World Health Organization Quality of Life Assessment (WHOQOL) (1998). The World Health Organization Quality of Life Assessment (WHOQOL). Development and psychometric properties. Soc. Sci. Med..

[b0130] Weight C.J., Sellon J.L., Lessard-Anderson C.R., Shanafelt T.D., Olsen K.D., Laskowski E.R. (2013). Physical activity, quality of life, and burnout among physician trainees: the effect of a team-based, incentivized exercise program. Mayo Clin. Proc..

[b0135] WHO (2018). Global Strategy on Diet, Physical Activity and Health [WWW Document].

[b0140] WHO, 2005. WHO STEPwise Approach to NCD Surveillance – Country-Specific Standard Report, Saudi Arabia.

[b0145] WHO, n.d. Body mass index - BMI [WWW Document]. URL http://www.euro.who.int/en/health-topics/disease-prevention/nutrition/a-healthy-lifestyle/body-mass-index-bmi.

[b0150] World Health Organization, n.d. Global Physical Activity Questionnaire (GPAQ) Analysis Guide [WWW Document]. URL https://www.who.int/ncds/surveillance/steps/resources/GPAQ_Analysis_Guide.pdf.

